# Biomarker candidates for progression and clinical management of COVID-19 associated pneumonia at time of admission

**DOI:** 10.1038/s41598-021-04683-w

**Published:** 2022-01-12

**Authors:** Joan Calvet, Antoni Berenguer-Llergo, Marina Gay, Marta Massanella, Pere Domingo, Maria Llop, Ester Sánchez-Jiménez, Marta Arévalo, Jorge Carrillo, Néstor Albiñana, Gianluca Arauz-Garofalo, Cristóbal Orellana, Juan Francisco Delgado, Alejandra Serrano, Artur Llobell, Eduard Graell, María García-Manrique, Mireia Moreno, Carlos Galisteo, Enrique Casado, Noemí Navarro, Antoni Gómez, Silvia Garcia-Cirera, Menna Rusiñol, Ester Costa, Bonaventura Clotet, Marta Vilaseca, Julià Blanco, Jordi Gratacós

**Affiliations:** 1 Rheumatology Department, Parc Taulí Hospital Universitari, c/Parc Taulí S/N, Edifici VII Centenari Rheumatology Department, 08208 Sabadell, Spain; 2 Institut d’Investigació i Innovació Parc Taulí (I3PT), 08208 Sabadell, Spain; 3 Departament de Medicina, Universitat Autónoma de Barcelona (UAB), 08003 Barcelona, Spain; 4 Institute for Research in Biomedicine Barcelona (IRB Barcelona), The Barcelona Institute of Science and Technology (BIST), 08028 Barcelona, Spain; 5 IrsiCaixa AIDS Research Institute, Germans Trias i Pujol Research Institute (IGTP), Can Ruti Campus, UAB, 08916 Badalona, Catalonia Spain; 6 Infectious Diseases Department, Hospital de la Santa Creu i Sant Pau. Institut de Recerca Biomèdica del HSCSP Barcelona, Barcelona, Spain; 7 UDIAT Immunology Unit, Parc Taulí Hospital Universitari. Institut d’Investigació i Innovació Parc Taulí (I3PT), 08208 Sabadell, Spain; 8 University of Vic-Central University of Catalonia (UVic-UCC), 08500 Vic, Catalonia Spain

**Keywords:** Biomarkers, Infectious diseases

## Abstract

COVID-19 pathophysiology is currently not fully understood, reliable prognostic factors remain elusive, and few specific therapeutic strategies have been proposed. In this scenario, availability of biomarkers is a priority. MS-based Proteomics techniques were used to profile the proteome of 81 plasma samples extracted in four consecutive days from 23 hospitalized COVID-19 associated pneumonia patients. Samples from 10 subjects that reached a critical condition during their hospital stay and 10 matched non-severe controls were drawn before the administration of any COVID-19 specific treatment and used to identify potential biomarkers of COVID-19 prognosis. Additionally, we compared the proteome of five patients before and after glucocorticoids and tocilizumab treatment, to assess the changes induced by the therapy on our selected candidates. Forty-two proteins were differentially expressed between patients' evolution groups at 10% FDR. Twelve proteins showed lower levels in critical patients (fold-changes 1.20–3.58), of which OAS3 and COG5 found their expression increased after COVID-19 specific therapy. Most of the 30 proteins over-expressed in critical patients (fold-changes 1.17–4.43) were linked to inflammation, coagulation, lipids metabolism, complement or immunoglobulins, and a third of them decreased their expression after treatment. We propose a set of candidate proteins for biomarkers of COVID-19 prognosis at the time of hospital admission. The study design employed is distinctive from previous works and aimed to optimize the chances of the candidates to be validated in confirmatory studies and, eventually, to play a useful role in the clinical practice.

## Introduction

An emerging severe acute respiratory syndrome coronavirus 2 (SARS-CoV-2), causing coronavirus disease 19 (COVID-19), started in Wuhan (People’s Republic of China) and rapidly spread worldwide, negatively impacting daily human activities, straining the health care system and leading to a high mortality around the world^1^.

The specific pathophysiology of the SARS-CoV-2 infection is not yet fully understood. Different molecular, biological and immunological pathways have been suggested to describe both its aggressiveness and the specific body response to the viral infection^2–4^. Unfortunately, none of them has provided an explanation to all the COVID-19 features, and few of them have resulted in the proposal of new therapeutic strategies^5^. Approximately 80% of affected patients suffer from an asymptomatic to a mild form of the disease, while the remaining 20% of patients display a more severe form^6^. Although no current standardized treatment is available, admitted patients presenting a more severe form usually receive therapy based in three management options: glucocorticoids^7,8^; antiviral drugs, currently remdesivir^9,10^; and immunosuppressive drugs as interleukin-6 inhibitors (IL-6)^11,12^. Nevertheless, controversies remain about the efficacy and cost-benefits of these treatments, even after recent evaluation in clinical trials^13,14^. Thereby, the research on prognosis and treatment biomarkers seems a priority to identify patients at risk of critical disease evolution at the time of admission, and to provide them with therapies tailored to their clinical presentation.

Studies on SARS-CoV-2 infection using mass spectrometry (MS)-based high-throughput proteomics technologies are now leading the compilation of a large amount of protein data, which is likely to contribute to a complete understanding of the infection. In contrast to the traditional clinical approaches^15,16^, MS allows the detection of proteome changes at a global scale, simultaneously interrogating the expression levels of a high number of proteins according to a specific characteristic of interest, in an agnostic way and providing broad insights on the protein networks involved in the molecular pathways^17^. Recent results have evidenced that COVID-19 has a substantial impact on plasma and sera proteome^18–20^.This holds a promising potential for the prompt identification of biomarkers for the diagnosis, prognosis and/or therapeutic targeting in this rapidly growing pandemic which requires a quick scientific response. For studies such as these to succeed, a proper definition of the clinical outcome on a homogeneous and accurately designed set of patients is crucial^21^.

**Figure 1:**
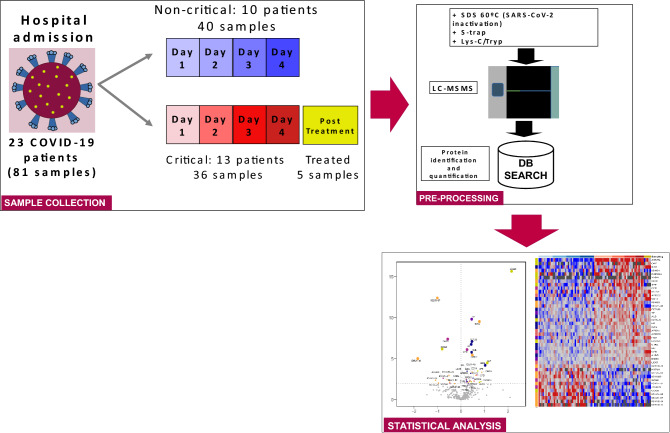
Study workflow: samples acquisition, proteome quantification and statistical analyses (figure created using R version 3.6.0, https://www.R-project.org/; and libreOffice version 6.4.7.2, https://www.libreoffice.org/).

This study aims to identify candidate proteins with the potential to be used in clinical practice as prognosis and clinical management biomarkers of the COVID-19 associated pneumonia at the time of admission. We conducted a MS-based proteomics experiment on plasma samples from 20 matched COVID-19 hospital patients before any prescription of COVID-19 specific treatment. Additionally, we profiled the plasma proteome of five patients before and after glucocorticoids and tocilizumab treatment, and assessed the changes induced by the therapy on our selected biomarker candidates (Fig. 1).

## Results

**Figure 2:**
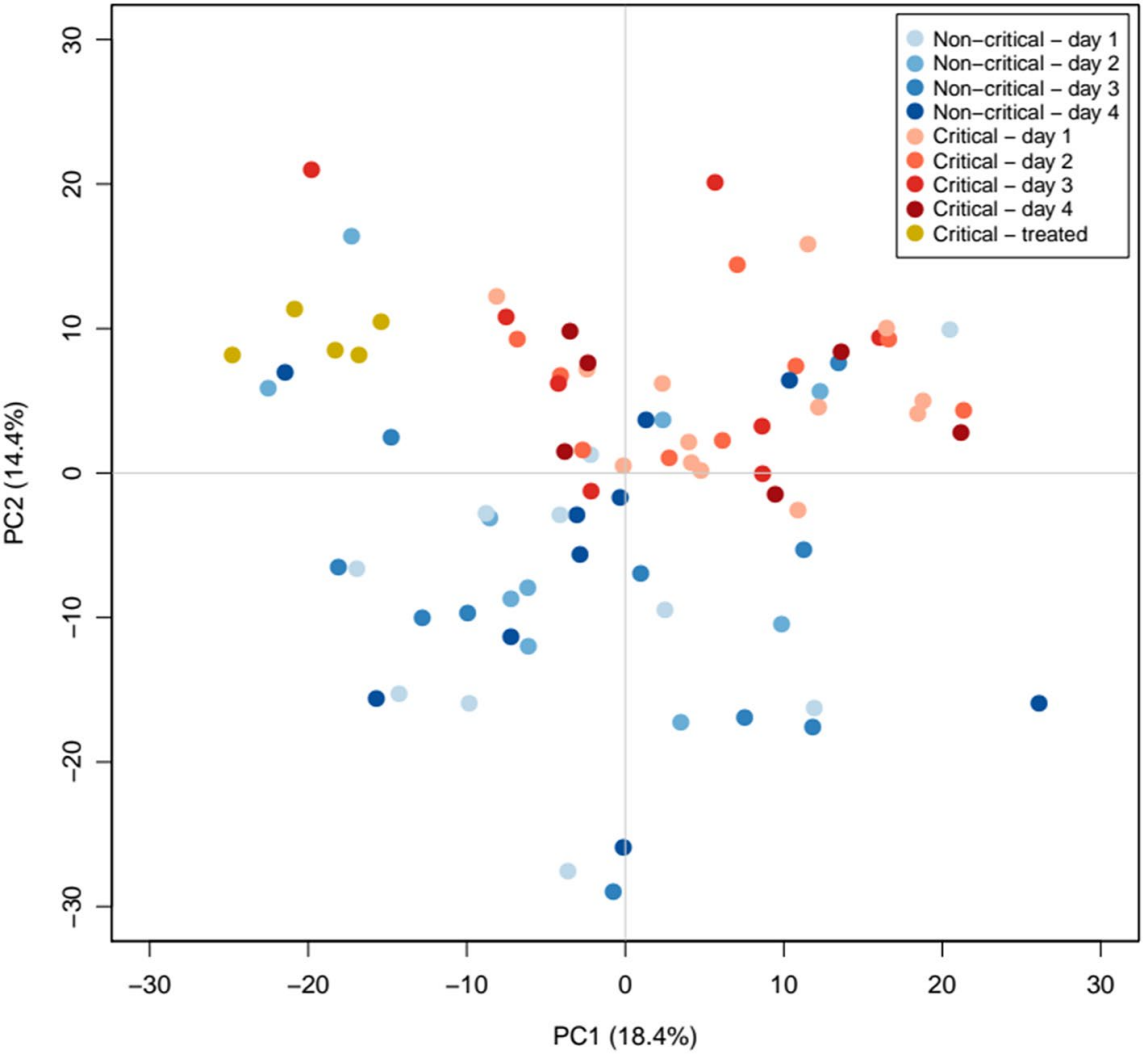
Principal component analysis of proteomic data at the peptide level. Dots represent the sample coordinates in principal components 1 and 2 derived from the 1.448 peptides m with available quantification for all samples in the study. Figures in the axes indicate the percentage of variance explained by the corresponding Principal Component. *PC1* principal component 1. *PC2* principal component 2. (Figure created using R, version 3.6.0, https://www.R-project.org/).

**Figure 3:**
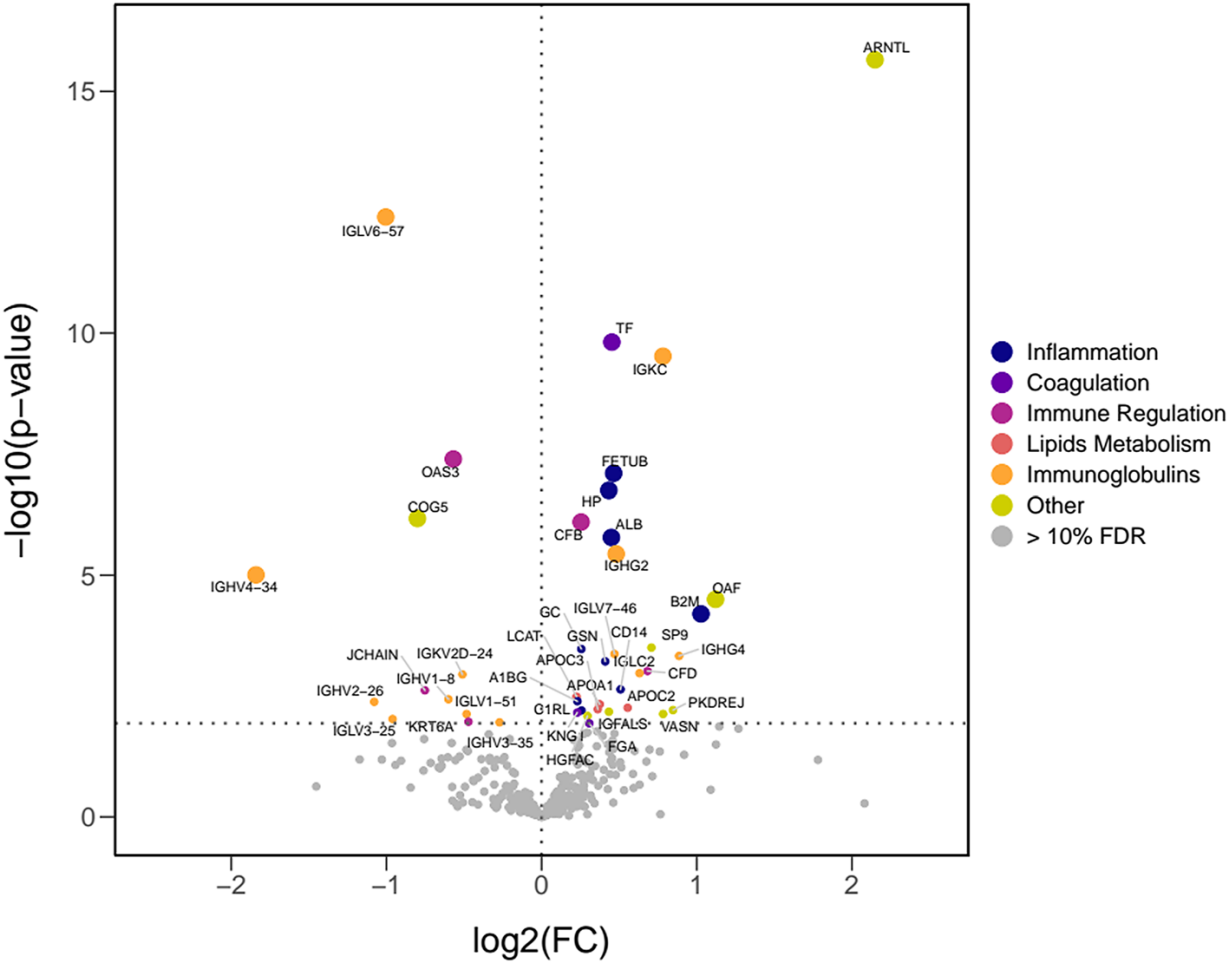
Volcano plot summarizing the results obtained from the differential expression analysis between critical and non-critical patients. X-axis represents the log2-transformed fold-change (FC). Y-axis shows the minus-log10-transformed p value associated to the protein in the comparison. Positive log2-fold-changes indicate over-expression in critical patients while negative log2-fold-changes represent over-expression in non-critical patients. Results were derived from a linear mixed-effects model fitted to each protein independently that included peptide and patient as random effects. Digestion batch, evolution group (critical/non-critical), blood extraction day and the interaction of the two latter were modelled as fixed effects. Comparisons were performed by averaging time point estimations within each evolution group. Statistical significance was assessed using a Wald test derived from the models. (Figure created using R version 3.6.0, https://www.R-project.org/).

We evaluated data from 13 COVID-19 patients with pneumonia who progressed to a critical condition during their hospital stay, together with 10 matched controls that experienced a favourable disease evolution (Fig. 1, Table 1). A total of 81 samples from 23 patients were available for the proteomics analyses (Supplementary Table 1), which identified 417 proteins across all samples. A Principal Component Analysis (PCA) performed on proteins quantified in all samples (172) clearly separated critical condition patients from those with favourable disease progression, even at the peptide level and before correcting by technical batches (Fig. 2, Supplementary Table 2). To identify which proteins were responsible for these differences, we performed a proteome-wise differential expression analysis on the 10 case–control sets where estimations for all time points were averaged within each evolution group (see details in Supplementary Methods). Up to 350 proteins provided quantifications in enough samples to perform such analysis, which identified 42 proteins differentially expressed between the two groups at 10% FDR (Table 2, Fig. 3, Supplementary Fig. 1; see Supplementary File 1 for all proteins results).

**Table 1:** Demographic and clinical parameters of patients included in the study.

	Non-critical N = 10	Critical N = 13	All N = 23
Gender—male	6 (60.0%)	8 (61.5%)	14 (60.9%)
Age at admission (years)	56.54 (41.94, 77.14)	61.09 (29.16, 69.91	60.59 (29.16, 77.14)
Hospitalization (days)	5.50 (4.00, 9.00)	15.50 (9.00, 31.00)	10.50 (4.00, 31.00)
Obesity	0 (0.0%)	1 (7.7%)	1 (4.3%)
Arterial hypertension	2 (20.0%)	3 (23.1%)	5 (21.7%)
Diabetes mellitus	0 (0.0%)	0 (0.0%)	0 (0.0%)
Hyperlipidaemia	1 (10.0%)	3 (23.1%)	4 (17.4%)
Glucocorticoid’s therapy	0 (0.0%)	13 (100.0%)	13 (56.5%)
Tocilizumab therapy	0 (0.0%)	12 (92.3%)	12 (52.2%)
Non-invasive mechanical ventilation	0 (0.0%)	9 (69.2%)	9 (39.1%)

Frequencies and percentages are used to summarize categorical variables, while median and the range (minimum and maximum values) are used for continuous variables.

**Table 2:** Proteins differentially expressed between critical and non-critical patients at 10% of false discovery rate (FDR).

Master protein accession	Protein description	Symbol	Critical vs non-critical			Post-treatment vs pre-treatment		
			Fold-change	p value	Adjusted p value	Fold-change	p value	Adjusted p value
O00327	Aryl hydrocarbon receptor nuclear translocator-like protein 1 OS = Homo sapiens OX = 9606 GN = ARNTL PE = 1 SV = 2	ARNTL	4.43	< 2.22e−16	< 2.22e−16	− 2.29	0.0341	0.0611
P06331	Immunoglobulin heavy variable 4–34 OS = Homo sapiens OX = 9606 GN = IGHV4-34 PE = 1 SV = 2	IGHV4-34	− 3.58	0.0000	0.0003	13.34	0.0000	0.0000
Q86UD1	Out at first protein homolog OS = Homo sapiens OX = 9606 GN = OAF PE = 2 SV = 1	OAF	2.17	0.0000	0.0008	− 1.50	0.0310	0.0571
A0A0B4J1V2	Immunoglobulin heavy variable 2–26 OS = Homo sapiens OX = 9606 GN = IGHV2-26 PE = 3 SV = 1	IGHV2-26	−2.11	0.0042	0.0520	− 1.37	0.1413	0.2117
P61769	Beta-2-microglobulin OS = Homo sapiens OX = 9606 GN = B2M PE = 1 SV = 1	B2M	2.04	0.0001	0.0016	1.73	0.0007	0.0017
P01721	Immunoglobulin lambda variable 6–57 OS = Homo sapiens OX = 9606 GN = IGLV6-57 PE = 1 SV = 2	IGLV6-57	− 2.01	0.0000	0.0000	− 1.19	0.5102	0.6062
P01717	Immunoglobulin lambda variable 3–25 OS = Homo sapiens OX = 9606 GN = IGLV3-25 PE = 1 SV = 2	IGLV3-25	− 1.95	0.0094	0.0847	− 1.13	0.8369	0.8778
P01861	Immunoglobulin heavy constant gamma 4 OS = Homo sapiens OX = 9606 GN = IGHG4 PE = 1 SV = 1	IGHG4	1.85	0.0005	0.0091	− 2.43	0.0000	0.0000
Q9NTG1	Polycystic kidney disease and receptor for egg jelly-related protein OS = Homo sapiens OX = 9606 GN = PKDREJ PE = 2 SV = 2	PKDREJ	1.80	0.0062	0.0667	− 1.59	0.0104	0.0212
Q9UP83	Conserved oligomeric Golgi complex subunit 5 OS = Homo sapiens OX = 9606 GN = COG5 PE = 1 SV = 3	COG5	− 1.74	0.0000	0.0000	1.33	0.0000	0.0001
Q6EMK4	Vasorin OS = Homo sapiens OX = 9606 GN = VASN PE = 1 SV = 1	VASN	1.72	0.0074	0.0704	−	−	–
P01834	Immunoglobulin kappa constant OS = Homo sapiens OX = 9606 GN = IGKC PE = 1 SV = 2	IGKC	1.72	0.0000	0.0000	− 2.17	0.0009	0.0023
P01591	Immunoglobulin J chain OS = Homo sapiens OX = 9606 GN = JCHAIN PE = 1 SV = 4	JCHAIN	− 1.68	0.0024	0.0351	− 1.36	0.0150	0.0292
P0CG40	Transcription factor Sp9 OS = Homo sapiens OX = 9606 GN = SP9 PE = 3 SV = 1	SP9	1.63	0.0003	0.0073	− 4.82	< 2.22e−16	< 2.22e−16
P00746	Complement factor D OS = Homo sapiens OX = 9606 GN = CFD PE = 1 SV = 5	CFD	1.61	0.0010	0.0168	− 1.12	0.2556	0.3386
P0DOY2	Immunoglobulin lambda constant 2 OS = Homo sapiens OX = 9606 GN = IGLC2 PE = 1 SV = 1	IGLC2	1.55	0.0011	0.0177	2.42	0.0013	0.0032
P0DP01	Immunoglobulin heavy variable 1–8 OS = Homo sapiens OX = 9606 GN = IGHV1-8 PE = 3 SV = 1	IGHV1-8	− 1.52	0.0037	0.0495	1.07	0.8190	0.8703
Q9Y6K5	2′-5′-oligoadenylate synthase 3 OS = Homo sapiens OX = 9606 GN = OAS3 PE = 1 SV = 3	OAS3	− 1.48	0.0000	0.0000	1.45	0.0002	0.0006
P02655	Apolipoprotein C-II OS = Homo sapiens OX = 9606 GN = APOC2 PE = 1 SV = 1	APOC2	1.47	0.0055	0.0644	1.34	0.1703	0.2438
A0A075B6R9	Probable non-functional immunoglobulin kappa variable 2D-24 OS = Homo sapiens OX = 9606 GN = IGKV2D-24 PE = 5 SV = 1	IGKV2D-24	− 1.42	0.0011	0.0179	− 1.50	0.0518	0.0878
P08571	Monocyte differentiation antigen CD14 OS = Homo sapiens OX = 9606 GN = CD14 PE = 1 SV = 2	CD14	1.42	0.0023	0.0350	1.89	0.0000	0.0000
P01701	Immunoglobulin lambda variable 1–51 OS = Homo sapiens OX = 9606 GN = IGLV1-51 PE = 1 SV = 2	IGLV1-51	− 1.40	0.0074	0.0704	− 1.01	0.9625	0.9685
P01859	Immunoglobulin heavy constant gamma 2 OS = Homo sapiens OX = 9606 GN = IGHG2 PE = 1 SV = 2	IGHG2	1.40	0.0000	0.0001	− 1.59	0.0022	0.0051
P02538	Keratin, type II cytoskeletal 6A OS = Homo sapiens OX = 9606 GN = KRT6A PE = 1 SV = 3	KRT6A	− 1.39	0.0107	0.0937	− 1.38	0.5156	0.6104
A0A075B6I9	Immunoglobulin lambda variable 7–46 OS = Homo sapiens OX = 9606 GN = IGLV7-46 PE = 3 SV = 4	IGLV7-46	1.38	0.0004	0.0087	− 2.25	0.0000	0.0000
Q9UGM5	Fetuin-B OS = Homo sapiens OX = 9606 GN = FETUB PE = 1 SV = 2	FETUB	1.38	0.0000	0.0000	1.11	0.2627	0.3453
P02787	Serotransferrin OS = Homo sapiens OX = 9606 GN = TF PE = 1 SV = 3	TF	1.37	0.0000	0.0000	− 1.14	0.0192	0.0371
P02768	Serum albumin OS = Homo sapiens OX = 9606 GN = ALB PE = 1 SV = 2	ALB	1.37	0.0000	0.0001	− 1.07	0.1371	0.2073
P35858	Insulin-like growth factor-binding protein complex acid labile subunit OS = Homo sapiens OX = 9606 GN = IGFALS PE = 1 SV = 1	IGFALS	1.35	0.0067	0.0687	1.07	0.2830	0.3674
P00738	Haptoglobin OS = Homo sapiens OX = 9606 GN = HP PE = 1 SV = 1	HP	1.35	0.0000	0.0000	− 1.24	0.0420	0.0735
P06396	Gelsolin OS = Homo sapiens OX = 9606 GN = GSN PE = 1 SV = 1	GSN	1.33	0.0006	0.0112	− 1.03	0.5348	0.6308
P02647	Apolipoprotein A-I OS = Homo sapiens OX = 9606 GN = APOA1 PE = 1 SV = 1	APOA1	1.30	0.0047	0.0563	1.07	0.2197	0.2972
P02656	Apolipoprotein C-III OS = Homo sapiens OX = 9606 GN = APOC3 PE = 1 SV = 1	APOC3	1.29	0.0059	0.0667	− 1.10	0.5091	0.6062
P02671	Fibrinogen alpha chain OS = Homo sapiens OX = 9606 GN = FGA PE = 1 SV = 2	FGA	1.24	0.0115	0.0961	1.39	0.0000	0.0000
Q04756	Hepatocyte growth factor activator OS = Homo sapiens OX = 9606 GN = HGFAC PE = 1 SV = 1	HGFAC	1.23	0.0081	0.0749	− 1.47	0.0045	0.0101
A0A0C4DH35	Probable non-functional immunoglobulin heavy variable 3–35 OS = Homo sapiens OX = 9606 GN = IGHV3-35 PE = 5 SV = 1	IGHV3-35	− 1.21	0.0111	0.0943	− 1.11	0.2434	0.3280
Q9NZP8	Complement C1r subcomponent-like protein OS = Homo sapiens OX = 9606 GN = C1RL PE = 1 SV = 2	C1RL	1.20	0.0063	0.0667	1.12	0.1735	0.2462
P02774	Vitamin D-binding protein OS = Homo sapiens OX = 9606 GN = GC PE = 1 SV = 2	GC	1.19	0.0003	0.0074	1.19	0.0006	0.0015
P00751	Complement factor B OS = Homo sapiens OX = 9606 GN = CFB PE = 1 SV = 2	CFB	1.19	0.0000	0.0000	1.89	0.0000	0.0000
P04217	Alpha-1B-glycoprotein OS = Homo sapiens OX = 9606 GN = A1BG PE = 1 SV = 4	A1BG	1.17	0.0041	0.0520	1.27	0.0000	0.0000
P01042	Kininogen-1 OS = Homo sapiens OX = 9606 GN = KNG1 PE = 1 SV = 2	KNG1	1.17	0.0069	0.0693	1.09	0.2509	0.3367
P04180	Phosphatidylcholine-sterol acyltransferase OS = Homo sapiens OX = 9606 GN = LCAT PE = 1 SV = 1	LCAT	1.17	0.0033	0.0455	1.23	0.0263	0.0489

The table also shows the results from the analysis comparing Treated and Not-treated samples for the same proteins. Results are derived from a linear mixed-effects model fitted to each protein independently that included peptide and patient as random effects. Digestion batch, evolution group (critical/non-critical), blood extraction day and the interaction of the two latter were modelled as fixed effects. Comparisons were performed by averaging time point estimations within each evolution group. Differences between treatment status were assessed in an analogous way, using a model that included treatment and batch of samples' digestion as fixed effects. Positive fold-changes indicate over-expression in critical patients or in Treated samples while negative fold-changes represent over-expression in non-critical patients or in Not-treated samples, respectively. Statistical significance was assessed using a Wald test derived from the models. The Benjamini–Hochberg method was used for control of the FDR (Adjusted p value).

Out of them, 12 showed a higher expression in patients with a more favourable disease progression (Fold-Changes: FCs from 1.21 to 3.58) which included: proteins related to immune regulation as 2′–5′-oligoadenylate synthase 3 (1.48 FC), Keratin type II cytoskeletal 6A (1.39 FC) and J Chain (1.68 FC); Conserved oligomeric Golgi complex subunit 5 (1.74 FC), and eight immunoglobulins related to immune response (IGLV6-57, IGHV4-34, IGKV2D-24, IGHV1-8, IGHV2-26, IGLV1-51, IGLV3-25 and IGHV3-35 from FC 1.21 to 3.58) (Table 2, Fig. 3, Supplementary Fig. 1).

Among the 30 proteins over-expressed in the critical condition group, (FCs from 1.17 to 4.43), we notably found a set of proteins related to the inflammatory cascade and immune modulation as Beta-2-microglobulin (FC = 2.04), Monocyte differentiation antigen CD14 (FC = 1.42), Fetuin B (FC = 1.38), Albumin (FC = 1.37), Haptoglobin (FC = 1.35), Gelsolin (FC = 1.33), Complement C1r subcomponent-like protein (FC = 1.20), Vitamin D-binding protein (FC = 1.19) and Alpha-1B-glycoprotein (FC = 1.17); proteins associated to immune modulation, inflammation and coagulation processes as Serotransferrin (FC = 1.37), Kininogen-1 (FC = 1.17), and Fibrinogen alpha chain (FC = 1.24); a set of proteins related to lipid metabolism, as Phosphatidylcholine-sterol acyltransferase (FC = 1.17) and a number of Apolipoproteins subtypes (APOA-I, APOC-II and APOC-III; FCs from = 1.29–1.47); Complement Factor B (FC = 1.19) and Complement Factor D (FC = 1.6), which are also associated to immune regulation; another set of immunoglobulins (IGKC, IGHG2, IGLV 7–46, IGHG 4, and IGLC 2; from 1.38 to 1.85 FC)_and, finally, other proteins with no apparent similarities at the signalling pathway level that included Aryl hydrocarbon receptor nuclear translocator like protein (FC = 4.43), Out at first protein homolog (FC = 2.17), Polycystic kidney disease and receptor for egg jelly-related protein (FC = 1.80), Vasorin (FC 1.72), Transcription factor Sp9 (FC = 1.63), Insulin-like growth factor-binding protein complex acid-labile subunit (FC = 1.35) and Hepatocyte growth factor activator (FC 1.23) (Table 2, Fig. 3, Supplementary Fig. 1).

Our analysis on pre- and post-treatment samples revealed massive changes in the patients' proteome 24 h after therapy with glucocorticoids and tocilizumab. Out of 322 proteins available for analysis, 139 showed over-expression while 52 displayed under-expression in treated compared to samples obtained before therapy, which were mainly involved in coagulation, complement, vascular cells, metabolism, inflammation and immunoglobulins (Supplementary Fig. 2, see Supplementary File 1 for complete results). Interestingly, the largest number of proteins with a different expression were related to complement factors and coagulation rather than to inflammatory cascade. Regarding the candidates for prognosis biomarkers (critical vs non-critical), two of the 12 proteins downregulated in critical patients showed an increase in their expression: OAS3 (1.45 FC), COG5 (1.33 FC). Furthermore, critical patients underwent a decrease in the expression on roughly a third (11 out of 30) of the proteins over-expressed in this group of subjects, which included ARNTL (− 2.29 FC), SP9 (− 4.82 FC), Hepatocyte growth factor (− 1.47 FC), Polycystic kidney disease and receptor for egg jelly-related protein (− 1.59 FC), Serotansferrin (− 1.14 FC), OAF (− 1.50 FC), Haptoglobin (− 1.24 FC), and a number of immunoglobulins as IGHG4, IGLV7-46, IGKC and IGHG2 (FCs from − 1.47 to − 2.43) (Table 2).

## Discussion

In our MS-based proteomics study, we found a total of 42 proteins differentially expressed in the plasma of critical and non-critical COVID-19 pneumonia patients at the time of admission. Furthermore, large-scale changes in the proteome of critical subjects were observed after their treatment with glucocorticoid and tocilizumab. Among them, two proteins (17%) under-expressed in critical patients underwent a significantly increase after therapy, while 11 proteins (37%) over-expressed in critical patients showed their expression decreased. Previous studies using a broad range of design and proteomics technologies have reported several proteins (27–93) as candidates of COVID-19 severity. Their profile of patients' plasma pointed to high specificity of several inflammation and immune modulators, in particular, pro-inflammatory signalling both upstream and downstream of IL-6, metabolic and immune dysregulation, and platelet and coagulation system activation^18–20,22,23^. Unfortunately, and despite these promising results, no sensitive biomarkers of COVID-19 prognosis have been successfully applied in clinical practice or trials to this date, and none of them had been evaluated for their usefulness in patients' clinical management.

Among the 12 proteins showing over-expression in non-critical patients, two of them stand out based on the magnitude of the differences observed between evolution groups and the changes induced in their expression by the therapy: Olygoadenilate synthetase 3 (OAS3) and Conserved oligomeric Golgi complex subunit 5 (COG5).

OAS3 is the highest molecular weight isoform among the OAS family, and its expression is activated by Type-1 and Type-3 interferons. Its combined high affinity for dsRNA and capability to produce 2-5As of sufficient length to activate RNase L, suggests that OAS3 might be a potent activator of RNase L, providing antiviral activity against RNA viruses. Previous studies identified an impaired type 1interferon (IFN) response associated with a persistent blood viral load and an exacerbated inflammatory response, so we hypothesize that subjects with high levels of OAS3 might develop a better response to SARS-CoV-2^24^. Additionally, type 1 IFN are crucial for immediate antiviral response by restricting replication and spread of the viruses. Therefore, an adequate production of IFN leads to an efficient T cell response while a delayed IFN response might cause the T cell exhaustion present in critical COVID-19 subjects^25^. Finally, there are no other IFN-induced proteins in the results of our analysis, suggesting that the activation of this antiviral pathway is specifically relevant in SARS-CoV-2 infection. COG5 is a subunit of oligomeric Golgi complex required for a normal function. Recently, a study reported a pathway specifically associated to the endoplasmic reticulum Golgi intermediate compartment as a relevant component for the SARS-CoV-2 viral production^26^. This finding suggests that COG5 might have a role in protection from COVID-19 infection via processes related to membrane transport. OAS3 and COG5 are two of the proteins that, while showing a lower expression in critical patients, their expression increased after treatment with glucocorticoids and tocilizumab. Taken together, these results suggest a role of these two proteins in COVID-19 severity and underscore their potential as biomarkers not only as for prognosis, but also in the decision making of patients' clinical management. On the other hand, and although also over-expressed in non-critical subjects, KRT6A and Immunoglobulin J Chain did not significantly change after treatment and, hence, their value as treatment biomarkers warrant further research. 

A larger set of markers^30^ were found over-expressed in critical condition. Regarding the inflammatory cascade, only Haptoglobin showed an expression decrease after treatment, while the rest of them remained over-expressed. This result could be explained by the short time interval between extraction of pre-and post-treatment samples (within 48–72 h), which might not be enough to observe a substantial decrease in the expression of the inflammatory factors. Confirmation of this observation, however, requires further research. 

Three proteins involved in the coagulation process were over-expressed in critical patients. Transferrin is an important clotting regulator, which might be related to thrombotic events in COVID-19^27^. Notably, Transferrin levels dropped after treatment, which may indicate an effect on the coagulation cascade. Kininogen-1 and fibrinogen alpha chain are involved in the stimulation of coagulation processes. Their decreased levels in post-treatment samples suggest an impact of therapy on the regulation of coagulation processes, as several pro-coagulant and anti-coagulant factors are found stimulated. Nevertheless, these observations need further research to draw definitive conclusions. 

Higher levels of a set of proteins related to lipid metabolism were also associated to critical condition, which also experienced an increase of expression after treatment. Although we hypothesize that their association might be related to inflammation, this point requires confirmation in specific studies.

Finally, we identified several proteins with no apparent shared signalling pathway. First and intriguingly we identified, ARNTL, which is part of biological clock helping to environment adaptation^28^ and showed the most extreme over-expression in critical patients. Previous studies have observed an increase of ARNTL levels in response to hypoxia conditions^29^. Interestingly, its expression significantly decreased after treatment, which could indicate potential not only for prognosis but also as a biomarker for clinical management. Other proteins displaying such expression decrease after treatment and, therefore, with potential value for clinical management were SP9, HGF and OAF.

Regarding immunoglobulins, our results suggests that effector functions of antibodies might be beneficial to control SARS-COV-2 infection. In non-critical patients, we observed an up-regulation of the classical complement activation pathways, while higher levels of alternative pathways were observed in subjects that reached a critical status^30^. Nevertheless, these associations could highly depend on the immunoglobulin subclasses and the mechanism of complement activation, so interpretation of these results should be taken with caution. It is noteworthy to highlight that, after treatment, all complement components significantly increased pointing to an immunological rather than anti-inflammatory effect of the treatment prescribed.

### Strengths and limitations

Our study has some strengths and limitations. Data collection was carried out at the end of the first COVID-19 incidence peak in Spain (late April 2020), which prevented us to recruit a higher number of patients. The small sample size analysed in this study determines the exploratory and provisional nature of its results. Therefore, a confirmatory study is required in a larger and independent set of patients to validate the reliability and applicability of these findings. This is especially true for the comparisons of pre- and post-treatment samples where, in spite of the paired design that enhance the statistical power of these analyses, only five samples per group were available. The exclusion criteria regarding age and previous pathologies also represent a limitation, as they avoid extrapolating the results to the general population. On the other hand, this patients’ selection also represents an advantage, as it allows to characterize the specific proteome associated with COVID-19 evolution, using a well-defined clinical outcome, in a highly homogeneous set of SARS-CoV-2 patients and, thus, increasing the chances to identify biomarkers of a mild to moderate magnitude. This study was carried out in a prospective cohort of patients with no previous COVID-19 specific treatment, although all patients with an unfavourable evolution ended up with some form of such therapies. Up to four blood samples from each patient in consecutive days were analysed, together with samples extracted before and after treatment with COVID-19 specific therapy for a limited number of patients, which ensured the robustness and consistency of the findings across the follow-up and allowed to obtain information about the candidates' potential as clinical management biomarkers. This design makes of the proteins identified in our study a good set of candidates for biomarkers of COVID-19 evolution, with potential to discriminate, at the time of admission, patients who could benefit from an early and more aggressive treatment. We reported unique candidates, especially three proteins, which had not been previously described as potential biomarkers for COVID-19 severity.

In conclusion, we propose a set of proteins as candidates for biomarkers of prognosis and clinical management of COVID-19 associated pneumonia patients. Since promising new therapies are ongoing, testing and validating the results reported in this work may have a critical impact on driving decision-making in clinical practice (see Supplementary Material for an extended Discussion section).

## Material and methods

### Patients and samples

Study's subjects were selected from a prospective cohort of patients systematically admitted to Hospital Universitari Parc Taulí between 14 and 28th April 2020. Patients had a confirmed diagnosis of COVID-19 based on viral sequence detection by reverse transcription-polymerase chain reaction (RT-PCR) of nasopharyngeal and/or oropharyngeal swab. All patients also showed SARS-CoV-2 pneumonia (defined as peripheral bilobar or bilateral infiltrates). Patients were included in the study before any prescription of COVID-19 specific treatment, as glucocorticoids, remdesivir or IL-6 inhibitors. However, all patients had started hydroxychloroquine, azithromycin and/or lopinavir/ritonavir treatment as per clinical practice at that time, previously to the time of inclusion. Recruitment stopped on 28th April due to Spain's favourable evolution of the pandemic at that time, and the subsequent drop in the number of COVID-19 hospitalized patients.

Exclusion criteria aimed to homogenize the patients' sample to optimize the chances for identification of clinically relevant markers in our proteomic study, and comprised potential confounding factors such as immunomodulatory treatments, active chemotherapy, age over 75 years, chronic renal failure, or patients under hemodialysis treatment, previous immunodeficiency, severe chronic obstructive pulmonary disease (FEV1 < 50%) and any opportunistic infection. All patients were remotely monitored to establish their condition status during follow-up as stable or as progression to critical COVID-19. Criteria for critical evolution was defined a priori as clinical features such as respiratory rate ≥ 30 breaths per minute with a PaO2 < 94% while on FiO2 ≥ 0.35, PaO2/FiO2 ratio < 200, or non-invasive mechanical ventilation or orotracheal intubation requirement. All patients who progressed to a critical disease condition (n = 13) were included in our study. For 10 of them, we selected a control (stable) patient matched by age, gender and, when possible, by classical cardiovascular risk factors, for a total of 23 individuals (Fig. 1, Table 1, Supplementary Table 1). According to the clinical management guidelines used at that time, all critical patients ended up under glucocorticoids and tocilizumab treatment. In contrast, none of these therapies were prescribed for any of the matched controls. All the subjects included in this study survived the infection process. This design and the characteristics of the patients selected for the study provide a suitable framework for the identification of prognostic biomarkers in the plasma proteome of COVID-19 patients, which could be used to discriminate patients at high risk of progressing to a critical condition of the disease and, thus, likely to benefit from specific treatments.

All patients systematically underwent four daily consecutive blood samples to get a complete display of their plasma proteome during the first days after hospital ward. Samples collection was stopped when the patient progressed to a critical condition or glucocorticoid treatment was prescribed according to clinical practice. Additionally, we obtained blood samples from five patients after glucocorticoids and tocilizumab treatment, which enabled us to profile proteome changes induced by these treatments and to assess these changes specifically in the prognosis biomarker candidates. Plasma samples were centrifuged, diluted, processed and stored until proteomic procedures. Patients' clinical information is described in Table 1 (see further details in Supplementary Methods).

### Proteomics analysis

For the MS-based proteomic analysis, we used a standardized label-free approach workflow described as follows. In order to control and correct experimental variation sources, samples were processed in batches that included a complete set of case–control patients. Ten μl of deactivated plasma sample (diluted 1:1 in SDS 8% 0.1 M DTT and heated at 60 °C) were reincubated with 4% SDS 0.05 M DTT for 5 min at 95 °C followed to 30 min at 55 °C. Detergent removal free cysteine thiols alkylation with iodoacetamide and sample digestion with Trypsin/LysC was done using S-trap columns (S-Trap mini kit (10 × 100 – 300 μg), reference K02-mini-10, Protifi) according to the established manufacturer protocol. Digested plasma samples were dried and reconstituted with 1% formic acid, 3% acetonitrile in an aqueous solution. On-line nanoLC-ESI–MS/MS was performed using a Dionex Ultimate 3000 ultrahigh-pressure chromatographic system coupled to an Orbitrap Fusion Lumos Tribrid mass spectrometer (Thermo Scientific). The Advion TriVersa NanoMate (Advion Inc. Biosciences) was used as the nanospray interface. Sample injections (600 ng of protein on column) were carried out in a specific order previously assigned at random. The mass spectrometer was operated in data-dependent acquisition (DDA) mode. Database search was done with Proteome Discoverer v2.3.0.523 (Thermo Scientific) using Sequenst HT as a search engine and Minora Feature Detector node to extract the LC–MS peaks used for peptide and protein quantification. SwissProt Human (released 2020_06) and Swissprot SARS (released 2020_07) databases were used. Peptides with a False Discovery Rate (FDR) < 1% were considered as positive identifications with a high confidence level. Unique peptides (peptides that are not shared between different protein groups) were considered for further quantitative and statistical analysis (see Supplementary Methods for a more detailed description). For quality control purposes, contribution of technical and clinical sources to the variability of the data was evaluated using a principal components analysis (PCA).

Evolution groups differences in the proteomic data were assessed for each protein independently, using linear mixed-effects models fitted to the log2-transformed expression values. The models included the patient's disease evolution (critical/non-critical), the time point of blood extraction (day 1–day 4) and the interaction of these two terms. Batch of sample's' digestion was considered as covariate for statistical control and random intercepts were considered to model the sample's patient of origin and the feature effect (peptide + modification + charge) when needed. Differences between treatment status (glucocorticoids and tocilizumab) were assessed in an analogous way, using a model that included treatment and batch of samples' digestion as fixed effects. A 10% FDR threshold was set for statistical significance. Results were graphically represented using heatmaps and Volcano plots (see further details in Supplementary Methods).

### Ethics approval and consent to participate

The Local Ethical Committee approved this study at the Parc Taulí Hospital Universitari (2020/569,14 of April 2020). All patients were verbally informed, and a witness informed consent was obtained as per regulatory conditions for COVID-19 studies in Spain. All methods were performed following the relevant guidelines and regulations.

## Supplementary Information

Supplementary Information 1.

Supplementary Information 2.

## Data Availability

All data relevant to the study are included in the article or uploaded as supplementary information. All data is available for the authors upon request.
