# Supplementary material for: Biomarker candidates for progression and clinical management of COVID-19 associated pneumonia at time of admission
Source: Sci Rep. 2022 Jan 12;12:640. doi: 10.1038/s41598-021-04683-w (PMC8755735; doi:10.1038/s41598-021-04683-w)
Supplement: Supplementary file 2 — Supplementary Information 2. [file 41598_2021_4683_MOESM2_ESM.docx]

**SUPPLEMENTARY TABLES**

**Supplementary Table 1.** Number of blood extractions by hospitalization day and evolution

and treatment group.

|  | **Non Critical** | **Critical Pre-treatment** | **Critical Post-treatment** |
| --- | --- | --- | --- |
| **Day 1** | 10 | 13 | 0 |
| **Day 2** | 10 | 9 | 0 |
| **Day 3** | 10 | 8 | 3 |
| **Day 4** | 10 | 6 | 2 |

**Supplementary Table 2.** Results from a Principal Variance Component Analysis (PCVA) performed on the proteomic data for peptides quantified in all samples in the dataset (1.448 peptides from 172 proteins). In each cell, the upper percentage represents the contribution of the technical or clinical parameter to the Principal Component, while the lower percentage estimates how much this contribution represents within the total variance observed in the data. Injection order was categorized in deciles for this analysis. Only the first 10 Principal Components are showed. The last row (Total) shows the contribution of each parameter to the total variance in the data (aggregation of lower percentages across all Principal Components). PC: Principal Component.

|  | **Digestion batch** | **Injection order** | **Evolution-Treatment** | **Extraction day** | **Patient effect** | **Residual variance** | **Total** |
| --- | --- | --- | --- | --- | --- | --- | --- |
| **PC1** | 0.0% 0.0% | 2.1% 0.4% | 62.8% 11.6% | 0.0% 0.0% | 9.8% 1.8% | 25.2% 4.7% | 18.4% |
| **PC2** | 2.9% 0.4% | 0.0% 0.0% | 51.3% 7.4% | 0.0% 0.0% | 13.8% 2.0% | 32.0% 4.6% | 14.4% |
| **PC3** | 16.5% 1.7% | 0.0% 0.0% | 0.0% 0.0% | 3.2% 0.3% | 0.0% 0.0% | 80.2% 8.4% | 10.5% |
| **PC4** | 0.0% 0.0% | 0.0% 0.0% | 0.0% 0.0% | 0.0% 0.0% | 8.8% 0.6% | 91.2% 6.3% | 7.0% |
| **PC5** | 0.0% 0.0% | 0.0% 0.0% | 17.1% 1.0% | 0.0% 0.0% | 31.7% 1.8% | 51.2% 3.0% | 5.8% |
| **PC6** | 0.0% 0.0% | 15.0% 0.6% | 0.0% 0.0% | 1.7% 0.1% | 17.9% 0.8% | 65.4% 2.8% | 4.2% |
| **PC7** | 0.0% 0.0% | 4.6% 0.2% | 9.9% 0.4% | 0.0% 0.0% | 44.2% 1.6% | 41.3% 1.5% | 3.6% |
| **PC8** | 0.0% 0.0% | 0.0% 0.0% | 8.3% 0.2% | 0.0% 0.0% | 54.3% 1.6% | 37.5% 1.1% | 2.9% |
| **PC9** | 0.0% 0.0% | 0.1% 0.0% | 0.0% 0.0% | 0.0% 0.0% | 48.1% 1.2% | 51.8% 1.3% | 2.5% |
| **PC10** | 2.4% 0.1% | 3.8% 0.1% | 0.0% 0.0% | 0.0% 0.0% | 23.8% 0.5% | 70.0% 1.5% | 2.1% |
| **TOTAL** | 3.2% | 1.9% | 21.1% | 0.7% | 14.9% | 58.1% | 100.0% |

**SUPPLEMENTARY FIGURES**


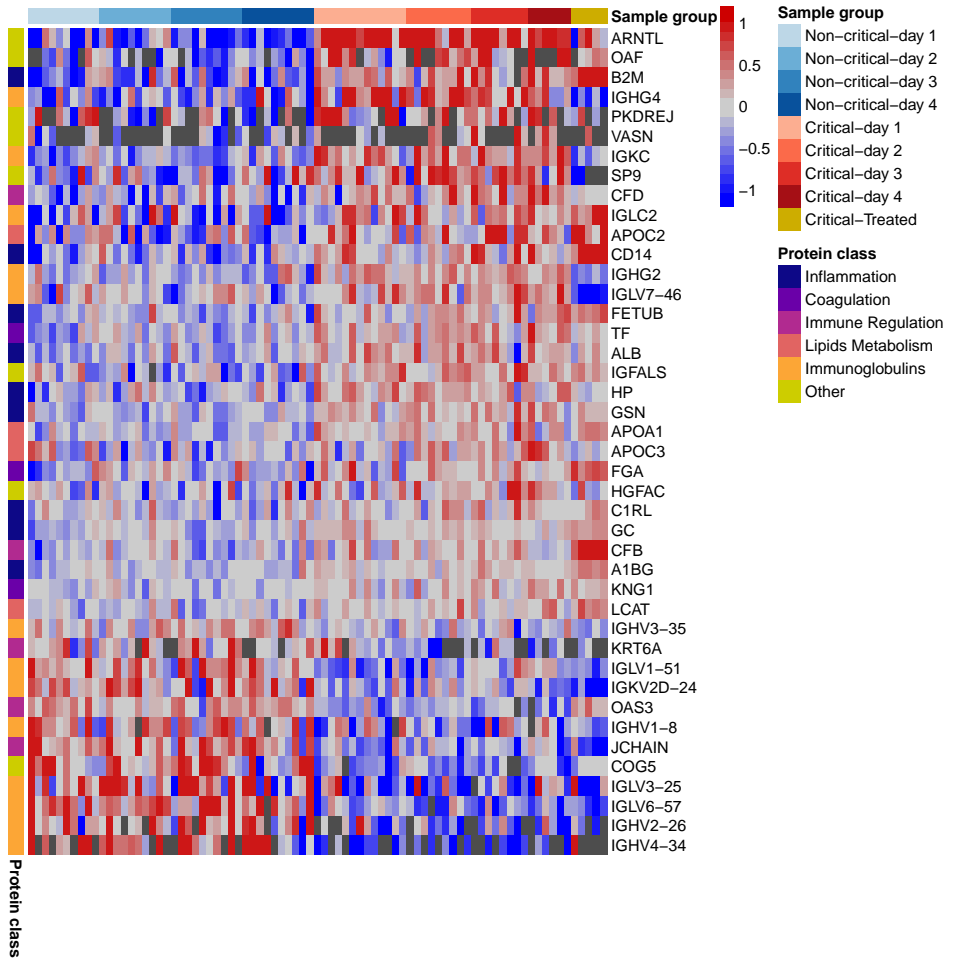


**Supplementary Figure 1.** Heatmap representing samples' expression values for proteins showing differential expression regarding evolution status (Critical vs Non-critical, 10% FDR) after correction by digestion batch and peptide effects. Expression values are centered protein-wise, truncated to percentiles 5% and 95% of the overall expression distribution, and represented in a colour gradient from blue (low expression) to red (high expression), where higher colour intensity matches to more extreme values in absolute value. Dark gray cells indicate samples with no available value in the corresponding protein. Proteins are showed in decreasing order according to their estimated fold-change in the comparison. (Figure created using R, version 3.6.0, https://www.R-project.org/).

**
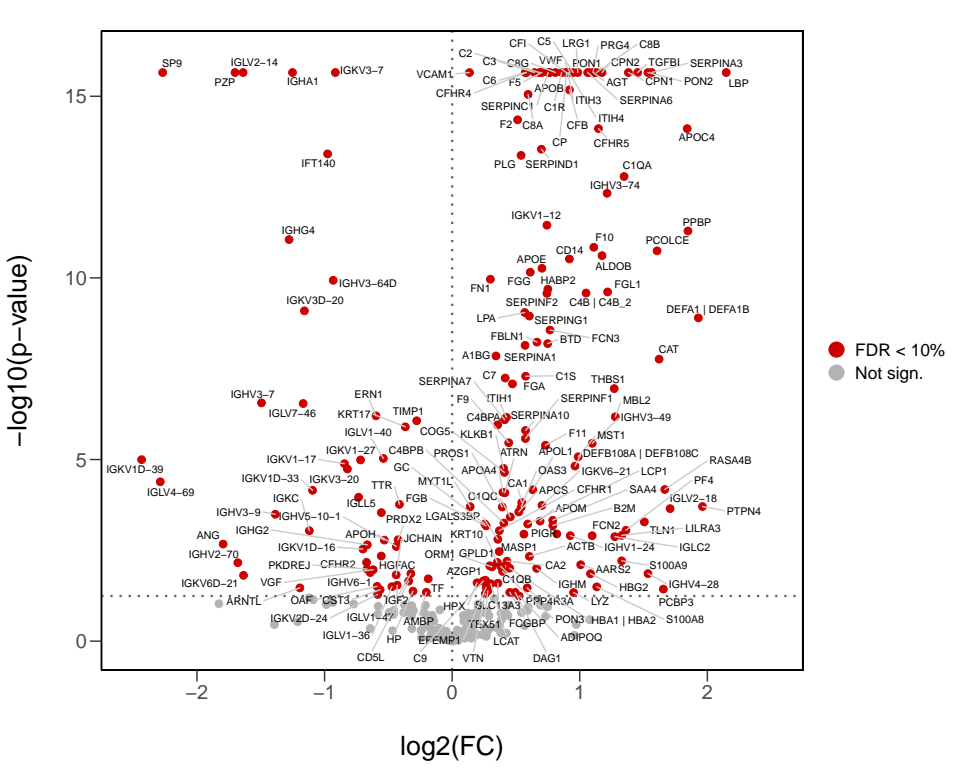
**

**Supplementary Figure 2.** Volcano plot summarizing the results obtained from the differential expression analysis between Treated and Non-treated samples. X-axis represents the log2-transformed fold-change (FC). Y-axis shows the minus-log10-transformed p-value associated to the protein in the comparison. Positive log2-fold-changes indicate over-expression in Treated samples while negative log2-fold-changes represent over-expression in Non-treated samples. Results were derived from a linear mixed-effects model fitted to each protein independently that included peptide and patient as random effects. Digestion batch and treatment status were modelled as fixed effects. Statistical significance was assessed using a Wald test derived from the models. (Figure created using R, version 3.6.0, https://www.R-project.org/).

**SUPPLEMENTARY METHODS**

**A. ASSESSMENTS**

All included patients systematically underwent four consecutive blood samples starting at the admission to the hospital ward. If patients progressed to a critical disease condition or glucocorticoid treatment according to clinical practice were prescribed before the four samples were drawn, only the samples collected previously these two events were used in the proteomic analysis (Figure 1). Plasma samples were centrifuged, diluted 1:1 in SDS 8% 0.1M DTT and heated at 60ºC according to the biocontainment procedures of the processing for SARS-CoV-2 positive samples and stored at -80ºC until the proteomic study procedure [a, b, c, d, e, f, g].

We also recorded the following information: sex, date of birth, date of symptoms onset, date of admission, date of confirmed diagnostic in the emergency room (positive RT-PCR from nasopharyngeal and/or oropharyngeal swab), and cardiovascular risk factors (arterial hypertension, hyperlipidaemia, diabetes, and obesity) defined by established diagnosis in medical history or taking active medication. Finally, the date of worsening (when applicable) and the discharge date from the hospital were collected. Patients, as per clinical practice requirement, underwent complete laboratory tests including all parameters typically related to COVID-19, such as total leucocyte, neutrophils, lymphocytes (cells × 109/L) counts, ferritin (ng/mL), CRP (mg/dL), D-Dimer (mg/mL) and lactate dehydrogenase (LDH, U/L).

**References.**

1. a) *H. F. Rabenau et al*, Stability and inactivation of SARS coronavirus. Med Microbiol Immunol (2005) 194: 1–6
2. b) *J.A Bow et al*, Virus inactivation by nuclei acid extraction regents. Journal of Virological Methods (2005) Vol 119(2): 195-198
3. c) *M.E. Darnell et al,* Inactivation of coronavirus that induces severe acute respiratory syndrome, SARS-CoV. Journal of Virological Methods 121 (2004) 85–91.
4. d) M.E. Darnell & D.R. Taylor. Evaluation of inactivation methods for severe acute respiratory syndrome coronavirus in noncellular blood products. Transfusion (2006) Vol 46:1770-1777
5. e) *T.J. Kochel et al*, Evaluation of TRIzol LS inactivation of viruses. Journal of ABSA International (2017) Vol 22(2): 52-55.
6. f) WHO (2003b) First data on stability and resistance of SAR coronavirus compiled by members of WHO laboratory network.

g) WHO (2004) Guidelines on viral inactivation and removal procedures intended to assure the viral safety of human blood plasma products.

**B. PROTEOMIC ANALYSIS**

For the differential quantitative proteomic MS-based analysis, we used a standardized label-free approach workflow detailed as follows.

**B.1. Plasma sample preparation**

In order to control and correct experimental variation sources, samples were processed in batches following a complete randomized block design that took into account the patients' characteristics (age, gender and cardiovascular risk factors).

Deactivated detergent-treated plasma samples were initially protein quantified with Pierce 660nm Protein Assay (Prod. #22662) and Ionic Detergent Compatibility Reagent (Prod.#22663).

300 µg (10 ul) of protein of each plasma sample were reincubated with the pre-made deactivation/denaturation/thiol reduction stock solution of 4% SDS 0.05 M DTT for 5 min at 95ºC followed to 30 min at 55ºC. 4 ul of 200 mM IAA (iodoacetamide) were added for free cysteine thiols alkylation and incubated at room temperature for 30 min in the dark. Detergent removal and sample digestion with Trypsin/LysC was done using S-trap columns (S-Trap mini kit (10x 100 – 300 μg), reference K02-mini-10, Protifi) according to the established manufacturer protocol. The digestion mixtures were eluted from the columns in three consecutive steps with 50 mM TEAB, 0.2% aqueous formic acid and 0.2% aqueous formic acid in 50% acetonitrile solutions. Finally, digested plasma samples were dried and reconstituted with 1% formic acid, 3% acetonitrile in an aqueous solution and stored at -20ºC until LC-MS analysis. Before injection to the nanoLC-MS/MS, samples were diluted 1:5.

**B.2. Nanoliquid chromatography electrospray ionization tandem mass spectrometry (nanoLC-ESI-MS/MS)**

On-line nanoLC-ESI-MS/MS was performed using a Dionex Ultimate 3000 ultrahigh-pressure chromatographic system coupled to an Orbitrap Fusion Lumos Tribrid mass spectrometer (Thermo Scientific). The Advion TriVersa NanoMate (Advion Inc. Biosciences) was used as the nanospray interface. Sample injections were carried out in a specific order in each sample batch to continue the statistics random design. We injected 600 ng of protein on column.

Peptide mixtures were loaded to a µ-Precolumn (300µm i.d x 5 mm, C18 PepMap100, 5 µm, 100 Å, C18 Trap column; Thermo Scientific) at a flow rate of 15 µL/min and separated using a C18 analytical column (NanoEase MZ HSS T3 column (75 µm × 250 mm, 1.8 µm, 100Å) (Waters)) with a flow rate of 250 nl/min and a 120 min linear gradient, from 3 to 35% B (A= 0.1% FA in water, B= 0.1% FA in CH3CN).

The mass spectrometer was operated in data-dependent acquisition (DDA) mode. In each data collection cycle, one full MS scan (350-1500 m/z) was acquired in the Orbitrap (1.2 x 10^5^ resolution setting and automatic gain control (AGC) of 1 x 10^5^. We used a dynamic exclusion of 60 s. The following MS2 analysis was conducted with a top speed approach with 3 s of cycle time. The most abundant ions were selected for fragmentation by high energy collisional dissociation (HCD) and detected in the ion trap. We used an HCD collision energy of 28%, an AGC target of 1 x 10^4^, an isolation window of 1.6 Da, a maximum ion accumulation time of 200 ms and a rapid ion scan rate. Spray voltage in the NanoMate source was set to 1.70 kV. RF Lens were tuned to 30%. The minimal signal required to trigger MS to MS/MS switch was set to 10,000. The mass spectrometer was working in positive polarity mode, and singly charge state precursors were rejected for fragmentation.

**B.3. Database searching**

Database search was done with Proteome Discoverer v2.3.0.523 (Thermo Scientific) using Sequenst HT as a search engine and Minora Feature Detector node to extract the LC-MS peaks used for peptide and protein quantification. SwissProt Human (released 2020_06) and Swissprot SARS (released 2020_07) databases were used. The search was run against the targeted and decoy databases to determine the false discovery rate (FDR). Search parameters included trypsin enzyme specificity, allowing for two missed cleavage sites, oxidation in M and acetylation in protein N-terminus as dynamic modifications and Carbamidomethyl in C as a static modification. Peptide mass tolerance was 10 ppm, and the MS/MS tolerance was 0.6 Da. Peptides and proteins with an FDR < 1% were considered as positive identifications with a high confidence level. Unique peptides (peptides that are not shared between different protein groups) were considered for further quantitative and statistical analysis.

**C. STATISTICAL METHODS**

For evolution groups comparisons, a mixed-effects linear model was fitted to the log2-transformed values of each protein separately, which included the patient's disease evolution (critical / non-critical), the time point of blood extraction (day 1 to day 4) and the interaction of these two terms. In these models, batch of sample's digestion was considered as covariate for statistical control and a random intercept was included in order to model the sample's patient of origin. In addition, when multiple features (peptides + modifications + charge) where available for the same protein, the feature was also modelled as a random effect in the model. Differences between treatment status (glucocorticoids and tocilizumab) were assessed in an analogous way, using a model that included treatment and batch of samples' digestion as fixed effects. Mixed-effects models were fitted using the *lmer* function from the *lme4* package [1]. Comparison between critical and non-critical patients was assessed by averaging the time point estimations within evolution groups, as day of extraction accounted for a low variability overall in the data (0.7%), according to a Principal Variance Components Analysis (PVCA) [2] (Supplementary Table 2). Mean differences, adjusted means and their corresponding standard errors were used to measure the magnitude of the effects. Statistical significance was assessed using the Wald tests derived from the models and the Benjamini-Hochberg method for control of the False Discovery Rate (FDR) [3]. A 10% FDR threshold was set for statistical significance. Data were graphically represented using Principal Component Analysis, and heatmaps; in the latter, expression values were centred protein-wise, truncated to percentiles 5% and 95% of the overall expression distribution, and subsequently represented in a colour gradient from blue (low expression) to red (high expression) where higher colour intensity matched to more extreme values in absolute value. For visualization purposes normalized protein intensities were corrected by feature (peptides + modifications + charge) and digestion batch using the coefficients derived from the model. Differentially expression results were also graphically represented in a Volcano plot, the log2-fold changes observed between sample groups was plotted against the corresponding minus log2(p-value) of the comparison. All these analyses were carried out using R [4].

**References.**

[1] Douglas Bates, Martin Maechler, Ben Bolker, Steve Walker (2015). Fitting Linear Mixed-Effects Models Using lme4. Journal of statistical Software, 67(1), 1-48. doi:10.18637/jss. v067.i01.

[2] Scherer A. Batch Effect and Experimental Noise in Microarray Studies: Sources and Solutions (2009), John Wiley & Sons.

[3] Yoav Benjamini and Yosef Hochberg. Controlling the False Discovery Rate: A Practical and Powerful Approach to Multiple Testing. Journal of the Royal Statistical Society. Series B (Methodological). Vol. 57, No. 1 (1995), pp. 289-300 (12 pages). Published by: Wiley. Journal of the Royal Statistical Society. Series B (Methodological). <https://www.jstor.org/stable/2346101>.

[4] R Core Team (2019). R: A language and environment for statistical computing. Version 3.6.0. R Foundation for Statistical Computing, Vienna, Austria. URL https://www.R-project.org/.

**EXTENDED DISCUSSION**

In our evaluation with label free quantitative proteomic analysis, a total of 42 proteins and immunoglobulins were differentially expressed in critical versus non-critical COVID-19 associated pneumonia patients at the time of admission. Previous studies using a broad range of design and proteomics technologies have reported several proteins (27 to 93) as candidates of COVID-19 severity. The proteome profiles in plasma capture the host response to COVID-19 infection and points to high specificity of several inflammation and immune modulators in particular, pro-inflammatory signalling both upstream and downstream of IL-6, metabolic and immune dysregulation, and platelet and coagulation system activation (1-5). Unfortunately, and despite these promising results, no sensitive biomarkers of COVID-19 prognosis or management have been applied in clinical practice to date.

Among the 12 proteins showing over-expression in non-critical patients, four proteins stand out based on the magnitude of the differences observed between evolution groups and their possible role in the physiopathology of the SARS-CoV-2 infection: Olygoadenilate synthetase 3 (OAS3), Conserved oligomeric Golgi complex subunit 5 (COG5), Keratin type II cytoskeletal 6A (KRT6A) and Immunoglobulin J Chain.

OAS3 is one of the proteins over-expressed in non-critical patients that aroused most of our interest. It is the isoform with the highest molecular weight in the OAS family and its expression is activated by Type-1 and Type-3 interferons. Its combined high affinity for dsRNA and its capability to produce 2-5As of sufficient length to activate RNase L suggests that OAS3 is a potent activator of RNase L promoting viral RNA degradation providing antiviral activity against several RNA viruses (6). Previous studies identified an impaired interferon (IFN) type 1 response associated with a persistent blood viral load and an exacerbated inflammatory response, so we hypothesize that subjects with high levels of OAS3 might develop an optimal response to SARS-CoV-2 (7). Additionally, IFN type 1 are crucial for immediate antiviral response by restricting replication and spread of the viruses. Therefore, an adequate production of IFN leads to an efficient T cell response while a delayed IFN response might cause the T cell exhaustion present in critical COVID-19 subjects (8). Finally, and interestingly, there are no other IFN-induced proteins in the results of our analysis, suggesting that the activation of this antiviral pathway is specifically relevant in SARS-CoV-2 infection. So, we consider of interest to explore OAS3 as a severity COVID-19 biomarker in further studies.

COG5 is another relevant protein that shows an increase (74%) in non-critical patients. COG5 is a subunit of oligomeric Golgi complex associated to glycosylation and traffic into Golgi complex and it is required for a normal Golgi function (9). Recently, a study reported a pathway specifically associated to the endoplasmic reticulum Golgi intermediate compartment as a relevant component for the SARS-CoV-2 viral production (10). Although this finding suggests that COG5 might have a role in protection from infection via processes related to membrane transport, its link with the COVID-19 infection is not fully understood and this pathway deserves a specific line of research.

KRT6A is a 39% over-expressed in non-critical patients. It is a type II keratin participating in the regulation of epithelial migration during wound repair. KRT6A also participates in antimicrobial host response, and a previous study related cytokeratins as a mediator of epithelial innate response (11). Although none related antiviral effects has been described, our findings suggest a role of KRT6A in antiviral response.

The other interesting protein showing a higher expression in non-critical patients is Immunoglobulin J Chain, which regulates polymer formation of immunoglobulin IgA and IgM. These immunoglobulins allow the transmembrane secretion of antibodies that are crucial for the first line defence against pathogens (12). In addition, a recent study identified the role of serum, and most specifically, mucosal IgA, in the early response against SARS-CoV-2 with neutralizing antibodies (13). Although no association with coronavirus or role in virus immunity has been described for J Chain (14), its implication in the secretory component and the results obtained in our study makes studying this protein worthwhile in the early phase of SARS-CoV-2 infection.

A larger set of markers (30) were found over-expressed in critical condition compared to non-critical patients. Out of them, nine proteins were related to inflammatory cascade, three were involved in coagulation processes, four had a role in lipid metabolism, two were linked to the immune regulation, five were immunoglobulins and the last seven did not appear to share a common pathway.

Regarding inflammatory cascade proteins, Fetuin B is a compound released by hepatocytes and related to lipid and glucose metabolism (15), and linked to chronic obstructive pulmonary disease (16). Strikingly, a previous study based on proteomic techniques related Fetuin B to COVID-19 severity in the opposite direction of association that the one derived from our analyses, as fatal patients showed lower levels of Fetuin B in that work (1). Differences in the classification of patients according their disease severity and in the scheme of blood extractions difficult the comparison between the two studies but, in any case, further research is needed to clarify the role of this marker in the evolution of SARS-CoV-2 infection. Low Albumin levels are associated to a high mortality rate (17). The levels of Albumin might increase in sepsis and inflammation situations, although its precise role it is not fully understood (18). We detected an up-regulation of albumin levels in critical COVID19 patients, but the methods used in our proteomic experiment do not allow us to estimate the absolute blood levels of this protein in our samples. Among its different functions, Haptoglobin plays a role in modulating many aspects of the acute phase response and immunity (19). The higher levels of expression found in critical patients for this protein suggest a role in the inflammatory cascade as it has been reported as biomarker candidate (2). Beta-2 microglobulin was also found over-expressed in critical patients, although it might be present simply as an immunity system regulator or as an acute phase reactant. Nevertheless, high levels of beta-2 microglobulin have been associated with low levels of CD4 and progression in HIV patients, while low abundance of CD4 cells have been reported to disease severity in previous COVID-19 studies (20). Vitamin-D-binding protein main function is involved in vitamin D transport and storage, and it has been also related to inflammation and immune activation. Previous studies have linked COVID-19 severity with vitamin D deficiency and several polymorphisms of Vitamin-D binding protein, pointing to a potential usefulness as prognosis COVID-19 biomarker (21, 22). Monocyte differentiation antigen CD14 is a plasma biomarker of monocyte-macrophage activation. Elevated levels of CD14 have been reported in measles and HIV patients with poor prognosis and an accentuated CD4+ T cells depletion (23, 24). A recent study related levels of CD14 to a need of hospital admission for COVID-19 infection suggesting an underlying role of monocyte and macrophage immune response in COVID-19 immunopathology. Moreover, a recent study proposes CD14 inhibition as a possible therapeutic approach to stop the inflammatory response associated to SARS-CoV-2, and has also been reported as biomarker for disease severity in a previous proteomic study (2). Plasma Gelsolin is an abundant blood protein and an important component of the innate human immunity. Gelsolin is depleted in an early phase of serious infections, independently from their viral or bacterial origin. It improves human protection against pathogens and modulates excessive inflammation avoiding damage associated to cytokine storm (25). Apparently in contrast to our findings (32% of expression increase in critical patients), previous proteomic studies reported down-regulation of Gelsolin in critical COVID-19 patient (2, 3). Of note, our study did not include controls or hospitalized subjects without oxygen requirements (WHO grade 3), and included WHO grade 4 as non-critical and WHO grades 5-6 as critical patients. Observing in detail the study of Messner et al, grade 5 patients showed higher levels of Gelsolin than grade 4 subjects, which is in agreement with our observation. Due to this consistent results, we suggest Gelsolin as a promising candidate for COVID-19 prognosis biomarker, while we consider of high interest to assess its levels in positive COVID-19 patient who do not require hospitalization in order to clarify its role in SARS-CoV-2 infection. Alpha-1B-glycoprotein is a protein which is prominent during the reactant acute phase of infections and is involved in neutrophil degranulation. Previous proteomic studies have found up-regulation of this protein in COVID-19 patients although, as in the case of Beta-2 microglobulin, these findings may probably be related to this reactant activity (2, 3). Complement C1r subcomponent-like protein mediates the proteolytic cleavages from preHA to Haptoglobin. Other functions related to this protein are linked to immune innate response and complement activation (26). To our knowledge no previous associations to SARS-CoV-2 nor other infectious diseases has been reported for complement C1r. All these considerations highlight the need for an exhaustive assessment and external validation of this set of markers to accurately evaluate their potential as prognosis biomarker of COVID-19 associated pneumonia.

Three proteins involved in the coagulation process were over-expressed in critical patients. Transferrin is an iron binding transport protein recently identified as an important clotting regulator, probably related to an interaction which reinforces the action of fibrinogen and additionally, mediates the antithrombin inhibition of coagulation (27). Therefore, transferrin levels might be related to thrombotic events in COVID19 patients (28). Kininogen-1 stimulates coagulation factors and acts as a mediator of inflammation (29). Fibrinogen alpha chain promotes coagulation and contributes to immune response in innate and T cell pathways (27). As COVID-19 disease produces a pro-thrombotic environment and no other coagulation-related molecules were found in our study, we consider of high interest to conduct further research on these three candidates and evaluate them as prognosis biomarkers.

Higher levels of a set of proteins related to lipid metabolism were also associated to critical condition in our study, which included Phosphatidylcholine-sterol acyltransferase, APOA-I, APOC-II and APOC-III. Some of them are components of HDL-cholesterol, a known modulator of inflammation and innate immune response (30, 31). Similarly, to Gelsolin, these results are in accordance with observations made in Messner et al in WHO grade 4 and 5 patients and, hence, further investigation are needed in longitudinal studies and different phases of the disease to understand the role of lipid metabolism in SARS-CoV-2 infection.

Finally, we identified a number of proteins with no apparent shared signalling pathway. First and intriguingly, we observed the most extreme over-expression in critical patients (> 4-fold-change) in Aryl hydrocarbon receptor nuclear translocator like protein, which forms part of biological clock helping the organism to environment adaptation (32). Previous studies have associated this protein to the regulation of cellular response to low oxygen levels with concomitant increased of ARNT levels in hypoxia conditions (33). We ignore the actual implication of this protein in the context of COVID-19 infection, but the results previously published suggest a role in an unfavourable COVID-19 evolution underscoring its interest for specific research and validation as prognosis biomarker. Vasorin is an extracellular cell surface glycoprotein mainly derived from vascular smooth muscle cells. An acute vascular injury produces a down-regulation of Vasorin modulating a fibroproliferative disorder promoted by TGF-β (34), so Vasorin interaction with TGF-β inhibits its vascular fibrogenic signalling. A previous study also reported that, a reduction of Vasorin, essentially related to a MMP-2 cleavage, amplifies the Angiotensin II and TGF-β1 signalling, promoting a fibrogenetic vascular environment (35). Regarding COVID-19, endotheliitis has been linked with SARS-CoV-2 infection (36) and a recent publication in critical patients associated a significantly decrease of Angiotensin-II levels with the development of acute respiratory distress syndrome (ARDS) (37). The role of Vasorin in COVID-19 is currently unknown but its association with Angiotensin II and endothelia disorders makes it deserve further investigation. Hepatocyte growth factor activator (HGF-A) is a protease which activates hepatocyte growth factor (HGF). COVID-19 disease has a pro-thrombotic environment characterized by high levels of thrombin, which could activate HGF-A (38). Previous studies have related HGF with COVID-19 severity (39). In the absence of other hypothesis, we suggest a link between HGF and coagulation factors to explain its association to critical COVID-19 subjects. Other proteins found with not known common pathway were: Out at first protein homolog, also named NS5ATP13 and associated to liver fibrogenesis with no other functions previously described; Transcription factor Sp9, involved in neuronal development; Insulin-like growth factor-binding protein complex acid labile subunit, which is implicated in protein interaction and in growth hormone control, and Polycystic kidney disease and receptor for egg jelly-related protein, which participates in fertilization. To our knowledge, no immune or infectious related functions has been attributed to these proteins, so their consideration as biomarker candidates for COVID-19 severity needs further research.

Regarding immunoglobulins and complement components, higher levels of IgG2 and IgG4 were observed in critical patients. Interestingly, these subclasses of IgG show lower effector functions than IgG1 or IgG3, which are the classes able to efficiently activate complement via the classical pathway. Conversely, IgG2 and IgG4 activates complement using almost exclusively an alternative mechanism and make use only marginally of the classical pathway. In addition, they are unable to sensitize mast cells, basophils and NK cells for killing (40). This observation suggests that effector functions of antibodies might be beneficial for control of SARS-COV-2 infection. Conversely, non-critical patients showed higher levels of J chain, a key component of IgM and IgA antibodies (see discussion above). As for the complement components, our results showed an up-regulation in non-critical patients of C1q, a member of the classical complement activation, while higher levels of components in alternative pathways (complements B and D) were observed in subjects that reached a critical status. The functional enrichment analysis showed a predominant over-represented profile of immunoglobulins humoral and immune response and complement activation in non-critical patient. These results are in agreement with the negative correlation between IgG1/3 responses and viral loads (41) and contribute to understand the role of the different immunoglobulins subclasses in disease pathogenesis and prognosis. Furthermore, our data is also consistent with a delayed humoral response in severe patients and with the suggested relevance of coordinated innate and adaptive responses in the control of viral replication (42). Nevertheless, and as discussed above, these associations could highly depend on the immunoglobulin subclasses and the mechanism of complement activation, so interpretation of these results should be taken with caution.

**References.**

1. Shu T, Ning W, Wu D, Xu J, Han Q, Huang M, et al. Plasma Proteomics Identify Biomarkers and Pathogenesis of COVID-19. Immunity. 2020;53(5):1108-22.e5.

2. Messner CB, Demichev V, Wendisch D, Michalick L, White M, Freiwald A, et al. Ultra-High-Throughput Clinical Proteomics Reveals Classifiers of COVID-19 Infection. Cell Syst. 2020;11(1):11-24.e4.

3. Shen B, Yi X, Sun Y, Bi X, Du J, Zhang C, et al. Proteomic and Metabolomic Characterization of COVID-19 Patient Sera. Cell. 2020;182(1):59-72.e15.

4. Schulte-Schrepping J, Reusch N, Paclik D, Baßler K, Schlickeiser S, Zhang B, et al. Severe COVID-19 Is Marked by a Dysregulated Myeloid Cell Compartment. Cell. 2020;182(6):1419-40.e23.

5. D'Alessandro A, Thomas T, Dzieciatkowska M, Hill RC, Francis RO, Hudson KE, et al. Serum Proteomics in COVID-19 Patients: Altered Coagulation and Complement Status as a Function of IL-6 Level. J Proteome Res. 2020;19(11):4417-27.

6. Ibsen MS, Gad HH, Thavachelvam K, Boesen T, Desprès P, Hartmann R. The 2'-5'-oligoadenylate synthetase 3 enzyme potently synthesizes the 2'-5'-oligoadenylates required for RNase L activation. J Virol. 2014;88(24):14222-31.

7. Hadjadj J, Yatim N, Barnabei L, Corneau A, Boussier J, Smith N, et al. Impaired type I interferon activity and inflammatory responses in severe COVID-19 patients. Science. 2020;369(6504):718-24.

8. Acharya D, Liu G, Gack MU. Dysregulation of type I interferon responses in COVID-19. Nat Rev Immunol. 2020;20(7):397-8.

9. Paesold-Burda P, Maag C, Troxler H, Foulquier F, Kleinert P, Schnabel S, et al. Deficiency in COG5 causes a moderate form of congenital disorders of glycosylation. Hum Mol Genet. 2009;18(22):4350-6.

10. Boson B, Legros V, Zhou B, Siret E, Mathieu C, Cosset FL, et al. The SARS-CoV-2 Envelope and Membrane proteins modulate maturation and retention of the Spike protein, allowing assembly of virus-like particles. J Biol Chem. 2020:100111.

11. Tam C, Mun JJ, Evans DJ, Fleiszig SM. Cytokeratins mediate epithelial innate defense through their antimicrobial properties. J Clin Invest. 2012;122(10):3665-77.

12. Johansen FE, Braathen R, Brandtzaeg P. The J chain is essential for polymeric Ig receptor-mediated epithelial transport of IgA. J Immunol. 2001;167(9):5185-92.

13. Sterlin D, Mathian A, Miyara M, Mohr A, Anna F, Claër L, et al. IgA dominates the early neutralizing antibody response to SARS-CoV-2. Sci Transl Med. 2021;13(577).

14. Epstein SL, Lo CY, Misplon JA, Lawson CM, Hendrickson BA, Max EE, et al. Mechanisms of heterosubtypic immunity to lethal influenza A virus infection in fully immunocompetent, T cell-depleted, beta2-microglobulin-deficient, and J chain-deficient mice. J Immunol. 1997;158(3):1222-30.

15. Meex RC, Hoy AJ, Morris A, Brown RD, Lo JC, Burke M, et al. Fetuin B Is a Secreted Hepatocyte Factor Linking Steatosis to Impaired Glucose Metabolism. Cell Metab. 2015;22(6):1078-89.

16. Diao WQ, Shen N, Du YP, Liu BB, Sun XY, Xu M, et al. Fetuin-B (FETUB): a Plasma Biomarker Candidate Related to the Severity of Lung Function in COPD. Sci Rep. 2016;6:30045.

17. Eckart A, Struja T, Kutz A, Baumgartner A, Baumgartner T, Zurfluh S, et al. Relationship of Nutritional Status, Inflammation, and Serum Albumin Levels During Acute Illness: A Prospective Study. Am J Med. 2020;133(6):713-22.e7.

18. Artigas A, Wernerman J, Arroyo V, Vincent JL, Levy M. Role of albumin in diseases associated with severe systemic inflammation: Pathophysiologic and clinical evidence in sepsis and in decompensated cirrhosis. J Crit Care. 2016;33:62-70.

19. Raju SM, Kumar AP, Yadav AN, Rajkumar K, Mvs S, Burgula S. Haptoglobin improves acute phase response and endotoxin tolerance in response to bacterial LPS. Immunol Lett. 2019;207:17-27.

20. Shi M, Taylor JM, Fahey JL, Hoover DR, Muñoz A, Kingsley LA. Early levels of CD4, neopterin, and beta 2-microglobulin indicate future disease progression. J Clin Immunol. 1997;17(1):43-52.

21. Ilie PC, Stefanescu S, Smith L. The role of vitamin D in the prevention of coronavirus disease 2019 infection and mortality. Aging Clin Exp Res. 2020;32(7):1195-8.

22. Speeckaert MM, Delanghe JR. Association between low vitamin D and COVID-19: don't forget the vitamin D binding protein. Aging Clin Exp Res. 2020;32(7):1207-8.

23. Sandler NG, Wand H, Roque A, Law M, Nason MC, Nixon DE, et al. Plasma levels of soluble CD14 independently predict mortality in HIV infection. J Infect Dis. 2011;203(6):780-90.

24. Gómez-Rial J, Currás-Tuala MJ, Rivero-Calle I, Gómez-Carballa A, Cebey-López M, Rodríguez-Tenreiro C, et al. Increased Serum Levels of sCD14 and sCD163 Indicate a Preponderant Role for Monocytes in COVID-19 Immunopathology. Front Immunol. 2020;11:560381.

25. Li GH, Arora PD, Chen Y, McCulloch CA, Liu P. Multifunctional roles of gelsolin in health and diseases. Med Res Rev. 2012;32(5):999-1025.

26. Wicher KB, Fries E. Prohaptoglobin is proteolytically cleaved in the endoplasmic reticulum by the complement C1r-like protein. Proc Natl Acad Sci U S A. 2004;101(40):14390-5.

27. Tang X, Zhang Z, Fang M, Han Y, Wang G, Wang S, et al. Transferrin plays a central role in coagulation balance by interacting with clotting factors. Cell Res. 2020;30(2):119-32.

28. McLaughlin KM, Bechtel M, Bojkova D, Münch C, Ciesek S, Wass MN, et al. COVID-19-Related Coagulopathy-Is Transferrin a Missing Link? Diagnostics (Basel). 2020;10(8).

29. Schmaier AH. The contact activation and kallikrein/kinin systems: pathophysiologic and physiologic activities. J Thromb Haemost. 2016;14(1):28-39.

30. Kumaraswamy SB, Linder A, Åkesson P, Dahlbäck B. Decreased plasma concentrations of apolipoprotein M in sepsis and systemic inflammatory response syndromes. Crit Care. 2012;16(2):R60.

31. Berbée JF, van der Hoogt CC, de Haas CJ, van Kessel KP, Dallinga-Thie GM, Romijn JA, et al. Plasma apolipoprotein CI correlates with increased survival in patients with severe sepsis. Intensive Care Med. 2008;34(5):907-11.

32. Gu YZ, Hogenesch JB, Bradfield CA. The PAS superfamily: sensors of environmental and developmental signals. Annu Rev Pharmacol Toxicol. 2000;40:519-61.

33. Wolff M, Jelkmann W, Dunst J, Depping R. The Aryl Hydrocarbon Receptor Nuclear Translocator (ARNT/HIF-1β) is influenced by hypoxia and hypoxia-mimetics. Cell Physiol Biochem. 2013;32(4):849-58.

34. Ikeda Y, Imai Y, Kumagai H, Nosaka T, Morikawa Y, Hisaoka T, et al. Vasorin, a transforming growth factor beta-binding protein expressed in vascular smooth muscle cells, modulates the arterial response to injury in vivo. Proc Natl Acad Sci U S A. 2004;101(29):10732-7.

35. Pintus G, Giordo R, Wang Y, Zhu W, Kim SH, Zhang L, et al. Reduced vasorin enhances angiotensin II signaling within the aging arterial wall. Oncotarget. 2018;9(43):27117-32.

36. Varga Z, Flammer AJ, Steiger P, Haberecker M, Andermatt R, Zinkernagel AS, et al. Endothelial cell infection and endotheliitis in COVID-19. Lancet. 2020;395(10234):1417-8.

37. Ozkan S, Cakmak F, Konukoglu D, Biberoglu S, Ipekci A, Akdeniz YS, et al. Efficacy of Serum Angiotensin II Levels in Prognosis of Patients With Coronavirus Disease 2019. Crit Care Med. 2021.

38. Shimomura T, Kondo J, Ochiai M, Naka D, Miyazawa K, Morimoto Y, et al. Activation of the zymogen of hepatocyte growth factor activator by thrombin. J Biol Chem. 1993;268(30):22927-32.

39. Janssen NAF, Grondman I, de Nooijer AH, Boahen CK, Koeken VACM, Matzaraki V, et al. Dysregulated innate and adaptive immune responses discriminate disease severity in COVID-19. J Infect Dis. 2021.

40. Carrillo J, Clotet B, Blanco J. Antibodies and Antibody Derivatives: New Partners in HIV Eradication Strategies. Front Immunol. 2018;9:2429.

41. Luo H, Jia T, Chen J, Zeng S, Qiu Z, Wu S, et al. The Characterization of Disease Severity Associated IgG Subclasses Response in COVID-19 Patients. Front Immunol. 2021;12:632814.

42. Sette A, Crotty S. Adaptive immunity to SARS-CoV-2 and COVID-19. Cell. 2021;184(4):861-80.
